# Anthropogenically driven spatial niche partitioning in a large herbivore assemblage

**DOI:** 10.1007/s00442-023-05342-9

**Published:** 2023-03-01

**Authors:** Nikhail Arumoogum, Jason P. Marshal, Francesca Parrini

**Affiliations:** Centre for African Ecology, School of Animal, Plant, and Environmental Sciences, Biology Building, University of the Witwatersrand, 1 Jan Smuts Avenue, Johannesburg, 2050 South Africa

**Keywords:** Niche, Partitioning, Ungulates, Anthropogenic, Disturbance

## Abstract

Understanding how human activity can influence species distributions and spatial niche partitioning between sympatric species is a key area of contemporary ecology. Extirpations of large mammalian populations, the result of a 15-year civil war, within the Gorongosa National Park in Mozambique, have been followed by an extended period of restoration. The species-specific recovery of these populations has provided an ideal system to identify how niche partitioning between coexisting species is altered as a consequence of extreme disturbance events. Here, we aimed to understand how distribution patterns of grazing herbivores, as well as spatial niche overlap between them, changed between the pre- and post-war scenarios. We focused on the following four grazer species: buffalo (*Syncerus caffer*); sable (*Hippotragus niger*); waterbuck (*Kobus ellipsiprymnus*); and zebra (*Equus quagga*). Using long-term aerial survey data, we quantified range size for each species, as well as spatial niche overlap between each species pair, for pre- and post-war periods. Range size of buffalo and zebra decreased drastically from the pre-war period; with both species inhabiting subsets of their historical distribution in the park. Sable and waterbuck have both colonised historically avoided habitat, with waterbuck doubling their pre-war range size. Spatial overlap between all four grazers pre-war was significantly high, indicating niche similarity; however, this decreased in the post-war period, with some species pairs displaying spatial niche dissimilarity. Our findings highlight how population responses to anthropogenic disturbance can result in significant alterations to species’ distributions, with consequences for patterns of niche similarity.

**Supplementary Information:**

The online version contains supplementary material available at 10.1007/s00442-023-05342-9.

## Introduction

Classic deterministic theories of ecology often view the degree of coexistence between species as a result of differences in ecological niche characteristics. For example, the competitive exclusion principle states that if two sympatric species fulfil equivalent ecological niches, then the species which has greater reproductive success will drive the other to extinction (Gause 1934; Hardin 1960). Building upon this, the principle of limiting similarity posits an upper limit to the extent of niche overlap between species in order for coexistence to be permitted (MacArthur and Levins 1967), whereas character displacement describes the process whereby natural selection favours the divergence of functional traits between sympatric species that are ecologically similar (Brown and Wilson 1956).

There are the following three key axes along which animal species often reduce excessive niche overlap: space, time and diet (Schoener 1974a, b, c). Species may partition spatial habitat use if they have differing resource requirements or physiological tolerances (Leal and Fleishman 2002), or if less competitive species are forced to persist in environments under less optimal conditions by competitively superior species (Jessopp et al. 2020). Where resources are limiting, dietary overlap can be facilitated either through spatial or temporal partitioning of food acquisition (Jessopp et al. 2020). In addition to food acquisition, spatiotemporal niche differences may also be attributed to interference competition or competition for habitats that reduce predation risk (Oriol-Cotterill et al. 2015; Munsch et al. 2016; Pudyatmoko 2019; Palmer et al. 2022). Examining how niche overlap between sympatric species is altered by external stressors can improve understanding of how resilient niche-based processes are to disturbance events.

Meta-analyses on carnivore communities suggest that increases in niche overlap between species are regular outcomes of both top-down (direct influence of humans or human activities and structures) effects as well as bottom-up (alteration of landscape characteristics and resource availability) effects (Sévêque et al. 2020). For example, fear of encounters with humans, as well as the provision of food resources by humans, may lead to greater spatiotemporal and trophic niche overlap between species in both large and meso-carnivore communities (Farris et al. 2017; Smith et al. 2018; Sogbohossou et al. 2018; Manlick and Pauli 2020). Limited evidence for the promotion of niche partitioning between carnivore species, through human modification resulting in greater habitat heterogeneity, has been found, although largely in Mediterranean systems (Pereira et al. 2012; Monterroso et al. 2014; Cruz et al. 2015). Within herbivore communities, both grazer and browser species have shown high selectivity for pastoral areas when available because of the availability of suitable forage as well as the relative scarcity of predators (Muchiru et al. 2008; de Jonge et al. 2022). Similarly, mass removal of large mammalian fauna in Gorongosa National Park, Mozambique, has resulted in a high degree of dietary overlap between sympatric grazer species (Pansu et al. 2019; Potter et al. 2022).

The relatively high species richness of grazing ungulates in African savannahs provide highly suitable systems to test hypotheses on niche partitioning. Partitioning of niche space between these coexisting species has often been framed around body size, as well as physiological and morphological traits that influence dietary niche breadth. In herbivore communities, large-bodied herbivores (termed megaherbivores when adult mass exceeds 1000 kg) may compete for food resources with medium-sized herbivores (mesoherbivores) between 50 and 500 kg, or facilitate coexistence with mesoherbivores via reducing the height of grass swards and staving off grass senescence (McNaughton 1976; Fritz et al. 2002; de Garine-Wichatitsky et al. 2004; Wegge et al. 2006). Regarding digestive physiology, hindgut fermenters such as zebra have shorter retention times for ingested food, providing them with an advantage in rate of ingestion of nutrient-poor foods over similar-sized ruminants when forage is readily available (Illius and Gordon 1992; Steuer et al. 2013).

Spatial niche partitioning between grazing ungulates may be driven predominantly by the risk of predation (Owen-Smith 2019a). For example, wildebeest prefer more open, short-grass, habitats that offer greater detectability of lions, whereas zebra and buffalo may inhabit areas ranging from high woody cover with tall grass to more open environments, as buffalo may defend themselves with their considerable bulk while zebra may use concealment to avoid detection as well as their hooves as protection (Martin and Owen-Smith 2016; Owen-Smith 2019a). In contrast, sable antelope may inhabit areas absent of other herbivore species that may be targeted as prey by lions (Owen-Smith et al. 2013). Conversely, increases in spatial niche overlap between herbivore species may be observed in order to form mixed-herds that dilute species-specific risks of predation (Schmitt et al. 2014). Spatial habitat preferences may have subsequent impacts on diet, where a grazer may have to eschew favourable habitat for habitat with less nutritious forage but lower predation risk (Owen-Smith et al. 2013). However, spatial niche characteristics may also be mediated by constraints imposed on dietary requirements, as zebra may be more flexible than wildebeest in spatially evading predation due to their greater tolerance for fibrous plant matter (Martin and Owen-Smith 2016).

Understanding how grazing ungulates partition niche space in southern Africa, and how this alters in response to drivers of change, is of growing importance. Increasingly variable temperature and rainfall patterns; subsequent scarcity of forage; as well as increased predation pressure have resulted in regional population declines in southern Africa (Ogutu and Owen-Smith 2003; Owen-Smith and Mills 2006; Owen-Smith et al. 2012). In Gorongosa National Park (hereafter Gorongosa), ungulate populations declined by > 95% in the late twentieth century as a result of the armed conflict between 1977 and 1992, whereby militia situated in Gorongosa hunted large mammals primarily for bushmeat (Stalmans et al. 2019). In the post-war interval, a period now twice as long as the duration of the disturbance event, large mammal populations have partially recovered and per unit area biomass is comparable with pre-war estimates [~ 9000 kg per km^2^; Stalmans et al. (2019)].

Population recoveries have been species-specific, however, with many species occurring at relative abundances that are substantially different from the pre-war scenario. Pre-war surveys from 1972 indicated the large mammal community of Gorongosa was dominated numerically by buffalo (*Syncerus caffer*; ~ 6000 individuals), with waterbuck (*Kobus ellipsiprymnus*; ~ 3000 individuals) and zebra (*Equus quagga*; ~ 2000 individuals) also relatively abundant while sable (*Hippotragus niger*; ~ 100 individuals) represented one of the rarest large herbivore species in Gorongosa (Stalmans et al. 2019). Most notably in the post war period (abundance estimates as of 2018), waterbuck have exploded in abundance by and order of magnitude (~ 50 000), while sable are substantially more abundant (~ 800) (Stalmans et al. 2019). In contrast, buffalo no longer represent the numerically dominant species (~ 1000), while zebra persist at numbers that put the species precariously close to extirpation within the park (~ 44) (Stalmans et al. 2019). Significantly, top-down predation pressure remains diffuse at the population level in Gorongosa, given the presence only of lion (*Panthera leo*) and wild dog (*Lycaon pictus*) in the park at time of study, both at relatively low abundances (Bouley et al. 2018; Atkins et al. 2019; Stalmans et al. 2019).

While the observed high dietary overlap between herbivores in the park (Pansu et al. 2019) heavily implies decreased trophic niche partitioning between sympatric species, the lack of comparable historical data hinders any direct comparison to pre-war interspecific dynamics. Systematic aerial surveys of Gorongosa recording the location of large mammals across the park, however, do allow for such a comparison. Our study aims to quantify how the armed conflict has altered the range size and spatial niche overlap of four sympatric grazer species in Gorongosa, namely buffalo, sable, waterbuck, and zebra. We chose to focus on these four species as they have been covered in both pre- and post-war surveys and represent species that currently persist at drastically different abundances than historical estimates (Stalmans et al. 2019). We hypothesized that changes in range size would mirror population trends (Gaston et al. 2000; Holt et al. 2002), such that species (namely waterbuck and sable) that have increased in abundance since the pre-war period would have larger range sizes, whereas species (buffalo and zebra) that have reduced in abundances now occupy smaller range sizes in the post-war period. We made two further, alternate, hypotheses regarding the changes in spatial niche overlap patterns that could be observed due to the civil war as follows:

**H1.** Species-specific shifts in distribution and range size due to the civil war may have altered patterns of spatial niche overlap, with the predicted reductions in buffalo and zebra range size leading to increased spatial niche dissimilarity.

**H2.** Conversely, due to the hyperabundance of waterbuck and greater abundance of sable, spatial niche overlap patterns from the pre-war period have remained the same, despite the predicted reductions in range size of buffalo and zebra.

## Methods and materials

### Study area

**Fig. 1:**
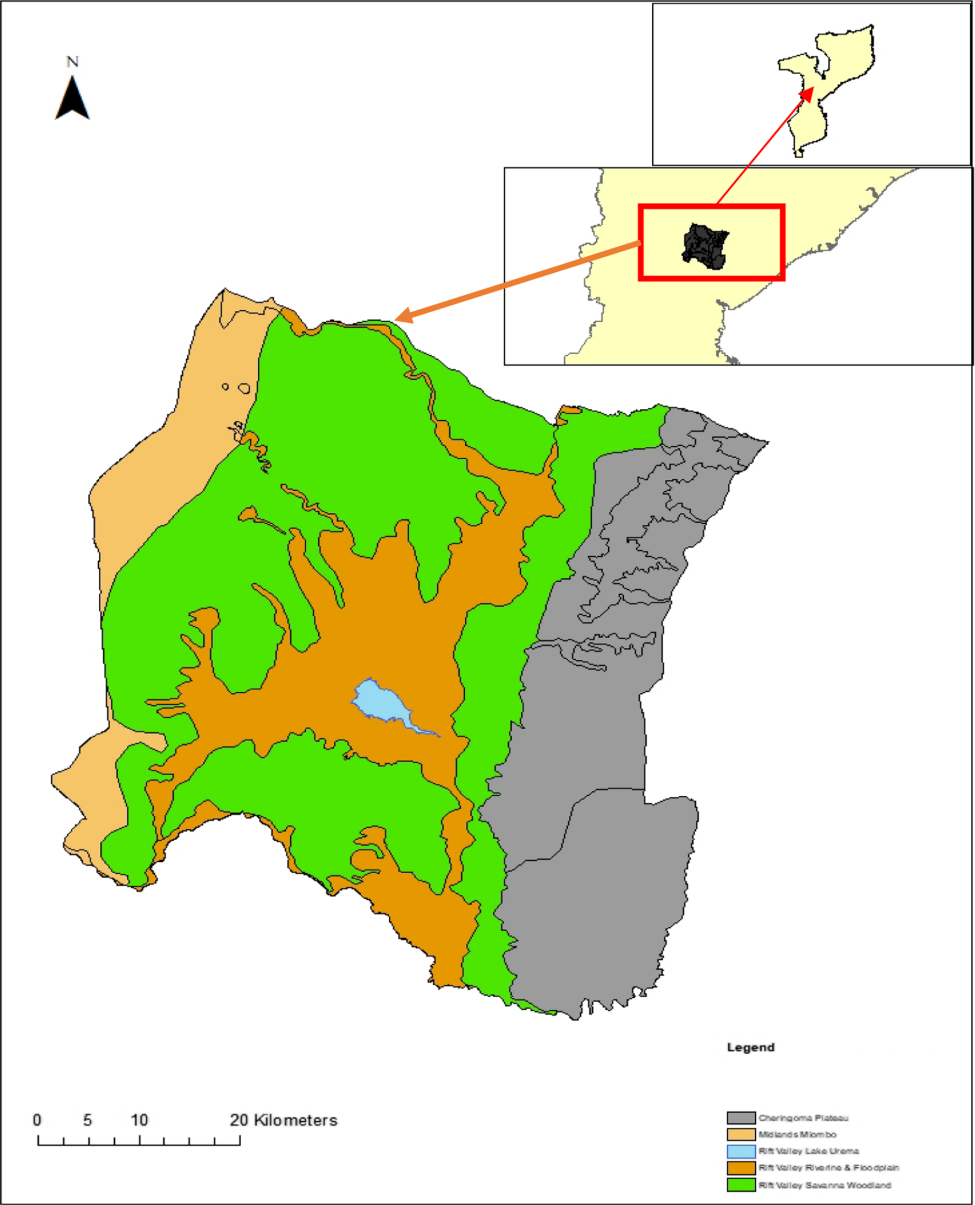
Extent of the Gorongosa National Park, with a representation of the broad-scale landscape types present within the park. Inset is the location of Gorongosa within the country of Mozambique

Gorongosa, encompassing an area of 3770 km^2^, is located in central Mozambique in the south of the Great Rift Valley (Fig. 1). The landscapes of the park are a patchy network of vegetation types, ranging from short grasslands and open floodplains to woodlands dominated by *Vachellia xanthophloea* (fever tree), *Senegalia* spp. (bushwillows) and *Combretum* spp. respectively (Stalmans and Beilfuss 2008; Mamugy 2016). *Hyphaene coriacea* (lala palm trees) are also distributed broadly throughout the park in the savanna-woodland mosaics. The centre of Gorongosa is characterised by a large open floodplain with a grass layer dominated by *Cynodon dactylon* and open surface water from Lake Urema.

The seasonal flooding of the floodplain landscape results in highly suitable forage for the herbivore community throughout the dry season (Tinley 1977). In conjunction with wet season flooding, fires (both of human and natural origin) influence vegetation structure in the dry season (Daskin et al. 2016). Dry season temperatures are cooler (averaging 26 ℃) than wet season temperatures (averaging 32 ℃), with approximately 90% of rainfall occurring during the wet season (Adesina et al. 2015).

### Study species

We selected four ungulate grazers that have substantially altered in relative abundance as a result of the armed conflict, namely, buffalo (*Syncerus caffer*); sable (*Hippotragus niger*); waterbuck (*Kobus ellipsiprymnus*); and zebra (*Equus quagga*). Georeferenced point localities for all species were collated from 19 aerial counts spanning the years 1969–2020 from the published dataset of Stalmans et al. (2019) and park management. Data were partitioned into pre-war (1969–1972; three surveys) and post-war (1994–2020; 13 surveys) years, including the most recent surveys either side of the Mozambican Civil War that took place between 1977 and 1992. Survey coverage of the park varied between years, ranging from 2.9 to 64.7% of the park area (Stalmans et al. 2019; Stalmans and Peel 2020). Aerial surveys in the pre-war period (1969–1972) counted abundance of herbivores within 500 m either side of a fixed wing aircraft, covering 64.7% of the park over a standardized area across all years (Table S1). Surveys between 2014 and 2020 were standardized to replicate the coverage of the pre-war surveys within a pre-assigned counting block for abundance, with additional parallel flight lines either side of the rift valley landscape, counting animals within 250 m either side of a helicopter (Table S1). Spatial coverage from surveys between 2014 and 2020 ranged from 49.9% of Gorongosa in 2014 to 58.9% in 2018. However, aerial surveys initiated soon after the end of the war (1994) until 2012 displayed more sporadic coverage of Gorongosa, ranging from 2.9% in 1994 to 23.8% in 2012. All but two of the 19 surveys were conducted in the late dry season (the months October and November) in order to allow for maximum detectability of animals. The two exceptions were the survey of 1970 which took place during the wet season in January, and the survey of 2012 which took place in the early dry season in May of that year. Although surveying during the dry season allows for maximum detectability, detection rates could not be accounted for given the lack of available data to do so, given that surveys were designed as game counts to measure abundance, and not to map spatial distributions.

Our data pooling approach does nullify the sensitivity and variation of changes in distribution between years within the pre-war and post-war scenarios; however, given the extremely low abundances of species in the immediate post-war years, data from surveys pre-2014 cannot be modelled in isolation. Moreover, the goal of our study was to analyse how spatial dynamics have altered now that a recovery period twice as long as the duration of the war has elapsed, and not to continuously monitor distribution shifts across shorter time steps, which are likely to be more heavily dependent on short-term sampling strategies..

To account for the inflation of spatial autocorrelation that can arise from sampling biases, we spatially filtered locality points within a radius of 1 km, for each species across each scenario, using the ‘spThin’ package (Aiello-Lammens et al. 2015) in R v. 3.4.0 (R Development Core Team 2014). This procedure identifies locality points that are clustered within a radius of 1 km or less and randomly removes all but one point. This ensures only one locality point per grid cell for analyses, reducing biases in estimates of environmental suitability for each species that may arise as artefacts of sampling strategy or coverage (Boria et al. 2014). This is crucial for ecological data collected over multiple sampling regimes where sampling intensity and procedure are variable, as is the case with the aerial wildlife surveys of Gorongosa (Stalmans et al. 2019). Spatially filtered datasets for each species were then used to build ecological niche models (ENMs) that estimate geographic distribution within the park.

### Environmental data

Bioclimatic data were downloaded from the WorldClim database v. 2 at a 30 arc-second resolution (~ 1 km; available at https://www.worldclim.org/data/index.html) (Fick and Hijmans 2017). These bioclimatic variables represent high-resolution, spatially-explicit distillations of long-term trends (1970–2000) in climatic factors useful for predicting contemporary species distributions (Fick and Hijmans 2017). Of the 19 bioclimatic variables available from the database, four (bio8, bio9, bio18 and bio19) were excluded due to large gaps in the data previously described by Allen et al. (2021). To reduce the dimensionality of the predictor dataset, we conducted a principal component analysis (PCA) on the 15 chosen bioclimatic variables in R v. 3.4.0 (R Development Core Team 2014). We used the first four principal components (PCs), which captured 97.32% of the variation contained in the original 15 variables (see Table S2 for summary of original variables, and Table S3 for component loadings).

All but two bioclimatic variables (bio3 and bio11) loaded high on PC 1, indicating that this component was representative of the synergistic effects of temperature and precipitation, and is likely a measure of productivity. Principal component 2 was a measure of minimum temperature extremes and temperature stability, whereas PC 3 was a measure of precipitation seasonality. Principal component 4 was a composite of isothermality and precipitation of the wettest month of the year.

### Ecological niche modelling

Maximum Entropy algorithms (Maxent) were used to calculate the geographic distribution of each selected species, in the Maxent v. 3.4 program (Phillips et al. 2006). Models built using a consensus from several different algorithms have been demonstrated to reduce error and provide more robust predictions (Araújo and New 2007; Diniz-Filho et al. 2009; Kaky et al. 2020). However, the Maxent algorithm has demonstrated comparable predictive performance to ensemble methods and does not require true absence data, fitting the presence-only data available in our study (Merow et al. 2013; Kaky et al. 2020). Most importantly, we chose to use Maxent instead of an ensemble approach as our main aim was to test hypotheses of niche similarity between geographic distributions, which is implementable using the Maxent algorithm in the ‘phyloclim’ R package (R Development Core Team 2014). Moreover, we did not project model predictions under different climatic scenarios that could have high levels of inherent uncertainty; thus the need for an ensemble approach here is not necessary (Araújo et al. 2019).

We calibrated models using nine candidate models for each species using three different values of regularization multipliers (1, 2, and 4) and three different combinations of feature classes (lqp = linear, quadratic, and product; lq = linear and quadratic; and q = quadratic). For all candidate models we used the first four PCs from the PCA conducted on the bioclimatic variables. For each species, we used 75% of the occurrence data for training models, and tested model predictions using the remaining 25% of occurrence data. Test and train data were randomly selected from the final dataset of occurrence points for each species. Pseudo-absence data were generated using 1000 randomly selected background points across the study area.

We did not use the linear feature class in isolation during model calibration, as species–environment relationships are often non-linear (Austin 2002). Regularization multiplier values below 1 were not used, as these fail to sufficiently punish candidate models for overfitting (Merow et al. 2013). We also did not use threshold or hinge features, as these are predominantly useful when constructing models where known physiological tolerance limits are experienced by the species over the study area (Merow et al. 2013). This entire process was repeated twice for each species, once using pre-war and once using post-war occurrence data, respectively.

We chose models based on Area Under the Curve of the Receiver Operating Characteristic (AUC) scores (Fielding and Bell 1997). The AUC score is independent of species’ prevalence across the study area and is thus useful in a cross-taxon approach using species with varied abundances (Allouche et al. 2006). However, the AUC may be misleading as it is dependent on the spatial extent of the study area (Lobo et al. 2008). While the True Skill Statistic (TSS) is suggested as a more accurate alternative (Allouche et al. 2006), measures based on analysing model performance on correctly predicted absences should be avoided when using presence-only data (Merow et al. 2013). Complementary log–log outputs of final models (representing probability of occurrence) were used for niche overlap analyses (Phillips et al. 2017). Binary model outputs reflecting presence/absence were obtained using the recommended method of maximising training sensitivity plus specificity, whereby the model selects a threshold that maximises the number of pixels that correctly predicted confirmed presences (Liu et al. 2013). We calculated range size for each species across each scenario using the binary outputs in R v. 3.4.0 (R Development Core Team 2014).

We assessed the importance of environmental variables on species’ distributions using the Maxent Jackknife test, which applies a permutation approach in constructing models using predictor variables in isolation to determine the information contributed by each variable to the regularized training gain.

### Niche overlap and similarity

Niche overlap for all possible species pairs, both pre- and post-war, was quantified using two indices – Schoener’s *D* and Hellinger’s *I* (Warren et al. 2008). Both indices work on a scale of 0–1, where 0 indicates no overlap in predicted habitat suitability between two species, and 1 indicates complete overlap. To distinguish whether niche overlap between two species was greater or smaller than would be expected by random chance, we conducted background similarity tests using the ‘phyloclim’ package in R v. 3.4.0 (R Development Core Team 2014). The background similarity test creates a distribution of expected *I* and *D* values by comparing the ENM of one species to a series of ENMs generated from random background points drawn from within the geographic region where both species are distributed (Warren et al. 2008). We compared observed *I* and *D* values to 99 expected *I* and *D* values to calculate two-tailed P-values. Geographic distributions were considered more similar than expected by chance if observed *I* and* D* values were greater than 97.5% of the expected *I* and* D* values. Geographic distributions were considered more dissimilar than expected by chance if observed *I* and* D* values were smaller than 97.5% of the expected *I* and* D* values. The background similarity test accounts for the underlying environmental conditions of the surrounding area and is not limited purely to the use of presence localities (Warren et al. 2010). Significant niche dissimilarity determined by this test indicates not only that species occupy distinct environmental niches, but also that these differences are not due to the environmental variation within the study area (Aguirre-Gutiérrez et al. 2015).

The background similarity test works in a fashion whereby two distinct distributions do not have to be reciprocally overlapping to be considered significantly similar. For example, distribution A may be largely, or even completely, encompassed by distribution B. As such, distribution A would be considered significantly similar to distribution B. However, the overlap in geographic space relative to the size of distribution B may be minimal, and hence distribution B may not be significantly similar to distribution A. This method allows us to investigate how pairwise species interactions have changed from both species’ perspectives across pre- and post-war scenarios. Moreover, we used the background similarity test to measure intraspecific niche changes and determine whether post-war distributions of species display niche similarity or dissimilarity to their pre-war distributions.

## Results

### ENM parameters and evaluation

Spatial filtering of locality points removed 95.33% of georeferenced localities for waterbuck in the post-war period due to spatial clustering (Table 1). However, the process still retained 1029 localities with which to build ENMs for this species in this period. Zebra in the post-war period had the least number of localities with which to build ENMs with (*n* = 45), however, this is well above the recommended number of 10 distinct localities (Schoeman et al. 2013). All other ENMs were built with a minimum of 110 localities.

**Table 1:** Number of georeferenced localities for each species across both pre- and post-war scenarios

	Number of pre-war localities used for ENMs		Number of post-war localities used for ENMs	
	Pre-filter	Post-filter	Pre-filter	Post-filter
Buffalo	298	260	206	110
Sable	114	112	755	407
Waterbuck	318	237	22,035	1029
Zebra	327	284	54	45

Number of pre-filter localities, as well as number of post-filter localities used to build ENMs are included

The best models, both pre- and post-war, were built with the regularization multiplier set to 1 and using a combination of linear, quadratic and product feature classes (Table S4-S7). This was observed for all species, except for zebra. The best pre-war model for zebra was constructed with the regularization multiplier set to 2, with a combination of linear, quadratic and product features. The best ENM for zebras post-war was constructed with the regularization multiplier set to 1, using a combination of linear and quadratic features.

Model AUC scores for training data were all > 0.5, indicating that models performed better than would be expected from a random fit (Table S4-S7). Pre-war training AUC scores for the best ENMs varied between species, ranging from 0.685 (zebra) to 0.794 (waterbuck). Training AUC scores for the best post-war ENMs ranged from 0.620 (waterbuck) to 0.835 (buffalo). Test AUC scores were almost identical to train AUC scores across all scenarios, with the largest difference between test and train AUC for the same model being 0.064, indicating that our models were not overfit.

### Environmental predictors

**Fig. 2:**
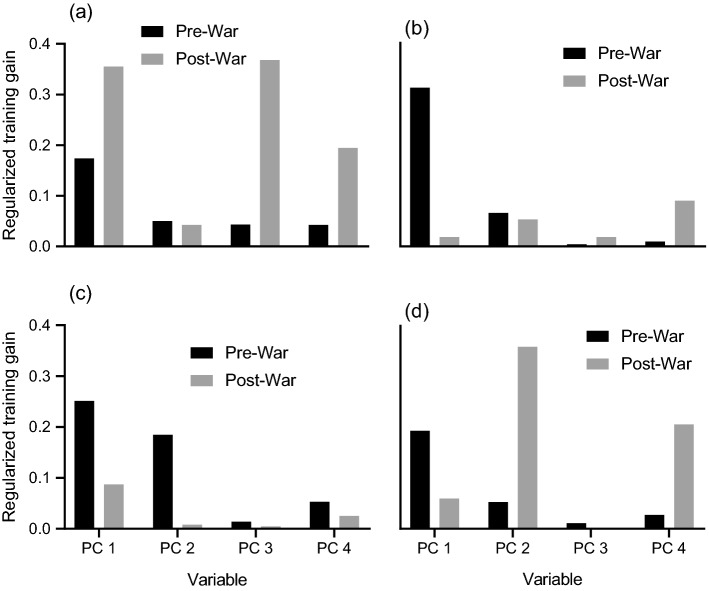
Isolated influence of environmental predictor variables on the regularized training gain for Maxent species distribution models for four species; **a** buffalo, **b** sable, **c** waterbuck, and **d** zebra. Black bars indicate influence of variables on pre-war distribution models, whereas grey bars indicate influence of variables on post-war distribution models

Principal component 1, representing productivity, was the most important driver of species distributions for all species in the pre-war period (Fig. 2). Buffalo in the post-war period were influenced predominantly by precipitation seasonality (PC 3) and productivity, while isothermality and precipitation of the wettest month (PC 4) was also an important predictor. Similarly, sable were influenced primarily by isothermality and precipitation of the wettest month, while zebra were predominantly influenced by minimum temperature extremes and temperature stability with a lesser influence of isothermality and precipitation of the wettest month. In addition to productivity (PC1), minimum temperature extremes and temperature stability (PC 2) was an important constraint on waterbuck distribution in the pre-war period. Waterbuck post-war distributions were still predominantly driven by productivity; however, all other species showed differing environmental preferences from their respective pre-war distributions.

### Range size

**Fig. 3:**
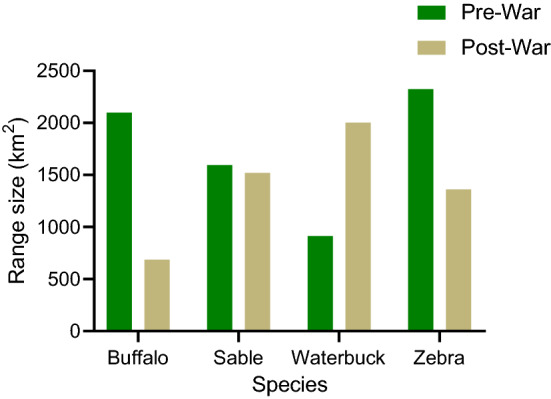
Pre- and post-war range size (in km^2^) of the four grazer species modelled for Gorongosa national park pre-war (1969–1972) and post-war (1997–2020). Range size was calculated using binary presence-absence outputs from Maximum Entropy modelling procedures

**Fig. 4:**
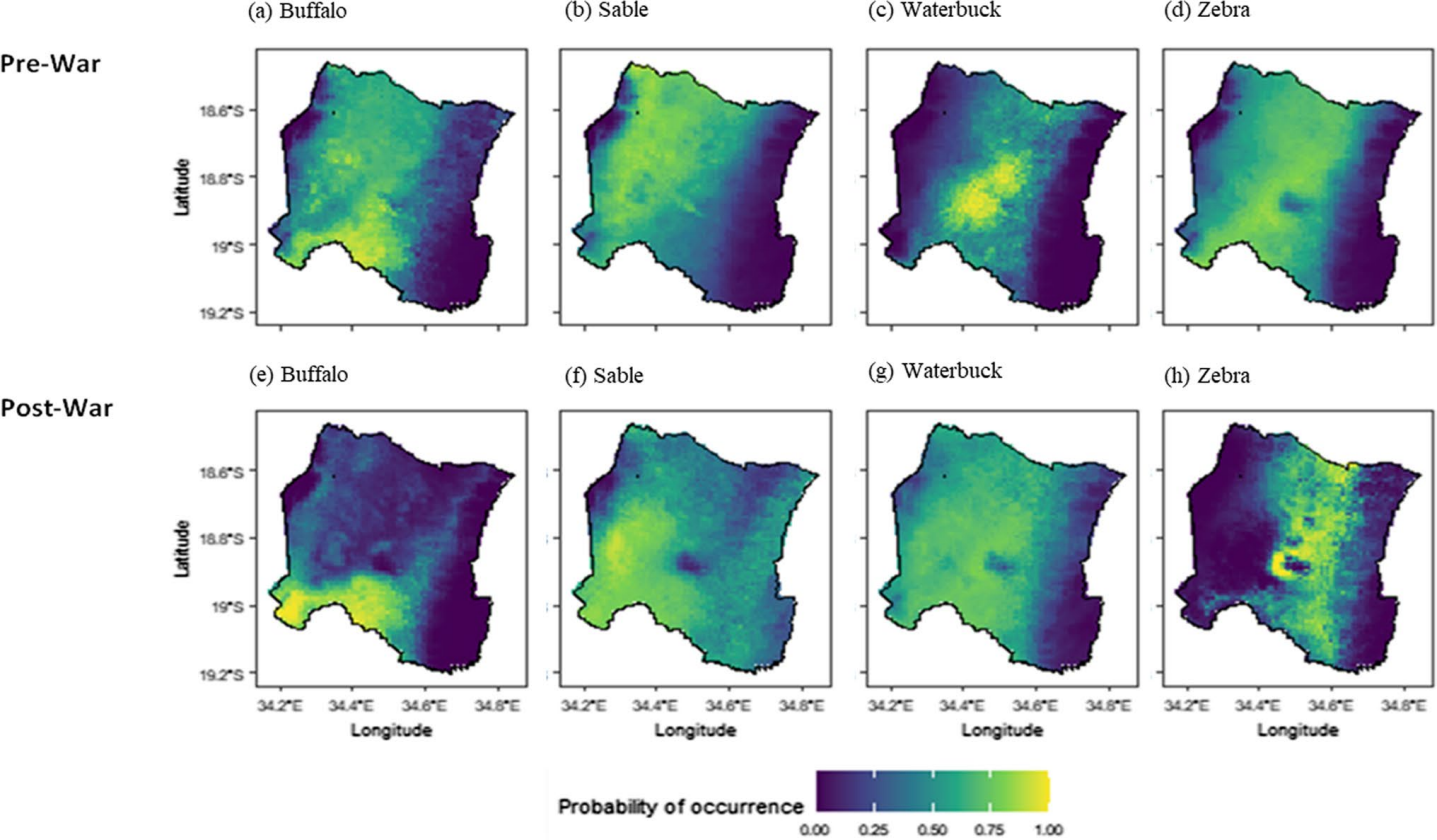
Pre-and post-war distributions within the Gorongosa National Park for four grazer species obtained by Maximum Entropy modelling. Blue areas indicate areas with a low probability of occurrence while yellow areas indicate high probability of occurrence. The top row represents pre-war spatial predictions for; **a** buffalo, **b** sable, **c** waterbuck, and **d** zebra. The bottom row is representative of the post-war spatial predictions for; **e** buffalo, **f** sable, **g** waterbuck, **h** zebra

Range size for all grazer species decreased between pre- and post-war years, with the notable exception of waterbuck (Figs. 3 and 4). Buffalo underwent the most drastic reduction in range size, from 2099.68 km^2^ (Fig. 3), covering over half the area of the park pre-war, to 686.40 km^2^ post-war, thus losing 65% of their range. Zebra had the largest pre-war range size for any of the grazer species in the study (2324.08 km^2^), decreasing by 42.71% to 1361.36 km^2^ post-war. In contrast, waterbuck had the smallest pre-war range size (913.44 km^2^) and have since more than doubled their post-war range size, the largest of any of the species considered in the study, to 2003.76 km^2^. Despite population estimates exceeding those pre-war, sable experienced minor reductions (4.64%) in range size, from 1594.56 km^2^ pre-war to 1520.64 km^2^ post-war.

### Intraspecific overlap and similarity

Zebra displayed the least amount of overlap between pre- and post-war distributions (*D* = 0.64; *I* = 0.87; Table 2) with buffalo displaying the second least (*D* = 0.67; *I* = 0.92). The two remaining species both showed relatively higher amounts of overlap: with similar overlap values for sable (*D* = 0.74; *I* = 0.93) and waterbuck (*D* = 0.78; *I* = 0.93).

**Table 2:** Results of the intraspecific background similarity tests

	Schoener’s *D*	Hellinger’s *I*
Buffalo	0.665*	0.922*
	(0.646, 0.731)	(0.876, 0.920)
	(0.476, 0.528)	(0.766, 0.814)
Sable	0.743*	0.927*
	(0.636, 0.679)	(0.865, 0.887)
	(0.740, 0.829)	(0.942, 0.975)
Waterbuck	0.782*	0.934*
	(0.580, 0.604)	(0.824, 0.840)
	(0.727, 0.792)	(0.920, 0.948)
Zebra	0.642*	0.871
	(0.614, 0.746)	(0.831, 0.906)
	(0.556, 0.619)	(0.833, 0.871)

The first set of brackets represents the confidence interval for the outputs based on species occurrence data for the pre-war distribution vs the background data for the post-war distribution. The second set of brackets represents the confidence interval for the outputs based on species occurrence data for the post-war distribution vs the background data for the pre-war distribution. Asterisks indicate significantly different overlap than expected by chance

Both sable and waterbuck pre-war distributions had greater overlap than expected by chance with their respective post-war distributions, however, post-war distributions for these two species had no significant overlap with their respective pre-war distributions (Table 2). The opposite was observed for both buffalo and zebra, where pre-war distributions showed no significant overlap with post-war distributions, however, post war distributions were significantly overlapped with pre-war distributions, indicating that these species inhabit subsets of their historical distributions.

### Interspecific niche overlap and similarity

Pre-war spatial overlap was relatively high for all pairwise species combinations (Tables 3 and 4). Schoener’s *D* values (Table 3) had a mean of 0.822 (± 0.062), ranging from 0.717 (waterbuck and sable overlap) to 0.902 (zebra and buffalo). Hellinger’s *I* values (Table 4) had a mean of 0.968 (± 0.021), ranging from 0.932 (waterbuck and sable overlap) to 0.989 (zebra and buffalo). The ubiquitously high overlap observed between grazers pre-war, was not evident in the post war period. Pairwise relationships including zebra and buffalo in particular, showed lower levels of spatial overlap. Schoener’s *D* mean values decreased to 0.636 (± 0.113) post-war, ranging from 0.472 (zebra and buffalo overlap) to 0.837 (waterbuck and sable overlap), whereas Hellinger’s *I* values had a mean of 0.872 (± 0.067), ranging from 0.758 (zebra and buffalo) to 0.968 (sable and waterbuck). Post-war overlap metrics showed a reversal of the extremes for pairwise-species interactions, whereby zebra and buffalo went from having the highest spatial overlap pre-war, to the lowest overlap post-war. In contrast, sable and waterbuck spatial overlap was lowest among all species pairs pre-war, while having the highest spatial overlap post war.

**Table 3:** Results of the post-war interspecific background similarity tests using Schoener’s *D*

	Buffalo	Sable	Waterbuck	Zebra
Buffalo	1	0.862	0.769	0.902
		(0.615, 0.690)*	(0.555, 0.616)*	(0.637, 0.704)*
		(0.655, 0.745)*	(0.667, 0.727)*	(0.660, 0.721)*
Sable	0.659	1	0.717	0.852
	(0.486, 0.521)*		(0.526, 0.643)*	(0.620, 0.732)*
	(0.738, 0.824)*		(0.619, 0.690)*	(0.631, 0.690)*
Waterbuck	0.674	0.837	1	0.827
	(0.498, 0.515)*	(0.777, 0.796)*		(0.641, 0.698)*
	(0.717, 0.795)*	(0.741, 0.786)*		(0.561, 0.619)*
Zebra	0.472	0.546	0.628	1
	(0.546, 0.634)*	(0.567, 0.606)*	(0.574, 0.596)*	
	(0.447, 0.581)	(0.691, 0.824)*	(0.685, 0.816)*	

Values above the diagonal represent the pre-war scenario and results below the diagonal represent the post-war scenario. Values in brackets represent 95% confidence intervals. The first set of brackets represents the confidence interval for the outputs based on species occurrence data for the species in the column vs the background data for the species in the row. The second set of brackets represents the confidence interval for the outputs based on species occurrence data for the species in the row vs the background data for the species in the column. Asterisks next to brackets indicate significantly different niche overlap than expected by chance, with similarity or dissimilarity inferred from whether the observed overlap value falls outside the lower or upper limit of the 95% confidence intervals

**Table 4:** Results of the post-war interspecific background similarity tests using Hellinger’s *I*

	Buffalo	Sable	Waterbuck	Zebra
Buffalo	1	0.983	0.950	0.990
		(0.851, 0.895)*	(0.808, 0.847)*	(0.848, 0.884)*
		(0.873, 0.928)*	(0.889, 0.918)*	(0.884, 0.917)*
Sable	0.892	1	0.932	0.984
	(0.780, 0.808)*		(0.785, 0.865)*	(0.837, 0.899)*
	(0.941, 0.973)*		(0.855, 0.892)*	(0.860, 0.892)*
Waterbuck	0.918	0.968	1	0.970
	(0.788, 0.802)*	(0.958, 0.965)*		(0.850, 0.884)*
	(0.914, 0.954)	(0.928, 0.945)*		(0.809, 0.849)*
Zebra	0.758	0.826	0.867	1
	(0.737, 0.852)	(0.916, 0.973)*	(0.891, 0.957)*	
	(0.826, 0.881)*	(0.840, 0.865)*	(0.846, 0.860)*	

Values above the diagonal represent the pre-war scenario and results below the diagonal represent the post-war scenario. Values in brackets represent 95% confidence intervals. The first set of brackets represents the confidence interval for the outputs based on species occurrence data for the species in the column vs the background data for the species in the row. The second set of brackets represents the confidence interval for the outputs based on species occurrence data for the species in the row vs the background data for the species in the column. Asterisks next to brackets indicate significantly different niche overlap than expected by chance, with similarity or dissimilarity inferred from whether the observed overlap value falls outside the lower or upper limit of the 95% confidence intervals

We found strong statistical support for ecological similarity between distributions for all possible species pairs pre-war (Tables 3 and 4). Greater than expected niche similarity was found for all possible species pairs, in a reciprocal fashion, i.e., sable displayed significantly high overlap when compared to buffalo background data and buffalo displayed significantly high overlap when compared to sable background data.

Post-war analyses revealed the erosion of stability in spatial ecological similarity of the grazer community (Tables 3 and 4). In particular, overlap between zebra and their sympatric grazers were significantly altered. Zebra-sable overlap was more dissimilar than would be expected by chance, in a reciprocal fashion, i.e., zebra distribution was significantly dissimilar to sable distribution and sable distribution was significantly dissimilar to zebra distribution. Zebra distribution was also significantly dissimilar to buffalo distribution (but buffalo distribution had no significant relationship with zebra distribution). Zebra distribution was significantly similar to waterbuck distribution; however, waterbuck distribution was significantly dissimilar to zebra distribution, indicating uncolonized suitable zebra habitat encompassed in the waterbuck distribution (Tables 3 and 4). Two other instances of significant ecological divergence in distribution were the sable-buffalo and buffalo-waterbuck relationships, where buffalo distribution was significantly overlapped with both sable and waterbuck distribution, respectively. Conversely, both sable and waterbuck distributions were significantly dissimilar to buffalo distribution. Only one interspecific pair was observed with reciprocal ecological similarity post-war (as opposed to all six pre-war): sable-waterbuck.

## Discussion

Using georeferenced localities to reconstruct period-specific ENMs, our results quantified intra- and interspecific changes to spatial niche dynamics across four grazer species. With the exception of sable, we found strong support for the hypothesis that species-specific changes in range size would mirror the direction of population trends, i.e., species that are more abundant in the post-war period relative to the pre-war period would display an increased range size. Intraspecific niche similarity tests also revealed expansion of the environmental niche space occupied by waterbuck and sable, whereas buffalo and zebra niches are currently nested within their respective historical niches. These species-specific changes have also altered interspecific spatial niche overlap patterns, such that the reciprocal niche similarity observed pre-war between all species pairs has been altered, with several species pairs now showing significant ecological dissimilarity between spatial niches in the post-war period.

### Intraspecific niche dynamics

The magnitude of increase in range size (119.36%) for waterbuck is unsurprising given the order of magnitude increase in abundance the species has undergone in the post-war era (Stalmans et al. 2019). While the influence of productivity on waterbuck distributions predominated both pre- and post-war environmental preferences (Fig. 2), niche similarity tests revealed that the ecological niche space inhabited by waterbuck pre-war was non-reciprocally ecologically similar to that inhabited post-war (i.e., post-war ecological niche was not significantly overlapped with pre-war niche). Combined with the relatively weaker constraints imposed by other environmental drivers in the post-war scenario (Fig. 2), all evidence indicates that waterbuck have expanded their spatial niche. Historically confined to the floodplains in the centre of Gorongosa (Fig. 4), they are now almost ubiquitous throughout the park. Post-war population growth of the waterbuck population imposed significant strain on the available foraging resources within the floodplain environments, necessitating use of historically uncolonized habitat (Becker et al. 2021).

The exact mechanisms that have permitted such rapid population growth of the species within Gorongosa may only be postulated given the paucity of monitoring schemes and coverage of data in the years immediately post-war. However, Stalmans et al. (2019) have postulated that the reliance of waterbuck on floodplain environments may have enabled them to evade much of the dangers that resulted in the declines of other species, as the substantial rise in water levels of the floodplain in the wet season render much of suitable waterbuck habitat inaccessible. Moreover, the short grass layer allows for easier detection of predatory threats from humans, and the lack of trees in this environment is not conducive for setting snares (Stalmans et al. 2019).

The colonization by waterbuck of novel habitat surrounding the floodplains, as well as the substantial population growth that necessitated this scenario, was likely facilitated by the almost complete extirpation of all terrestrial mammalian carnivores, as well as severe population declines of potential competitors such as buffalo and zebra (Bouley et al. 2018; Stalmans et al. 2019). Buffalo and zebra have both reduced substantially in range size, with zebra losing 41.42% of their range size while buffalo currently occupy a range that is less than a third of its original size (Fig. 3). The greater post-war influence of precipitation seasonality for buffalo and minimum temperature extremes and stability for zebra, indicate a departure from the pre-war predominance of productivity as a driver of habitat suitability across all species. Moreover, niche similarity tests suggest (Table 2) that post-war spatial niches for both species are ecologically similar to their pre-war spatial niches, but that pre-war spatial niches are not significantly overlapped with post-war niches. This, combined with the substantial reductions in range size, suggests that their current spatial niches are nested within their historical spatial niches, representing only a subset of the environmental conditions that once characterized the distributions of both species.

Sable represent an exception to our hypothesis regarding within species range size expansion being paired with positive population growth. Despite persisting at population densities that are unprecedented in Gorongosa, sable inhabited a slightly smaller area (4% decrease), comparable to their historical range size. The similar range size coupled with the non-reciprocal ecological similarity from pre-war to post-war niches, indicate shifts to new habitats. Highly suitable sable habitat occurred predominantly in the south in the post-war scenario, shifting from the pre-war period, where the northern and central parts of the park had the most suitable habitat for sable. It is likely that sable, like waterbuck, have dispersed into previously unoccupied habitats that became available after the loss of competitors such as buffalo and zebra which also feed on tall grasses, as well as predators such as lion (Chirima et al. 2013; Bouley et al. 2018).

It is unclear why sable have been one of the beneficiaries of population growth and subsequent range size expansion in the post-war period. Aerial surveys immediately post-war revealed that the severe population declines (90–99%) observed in other species extended to sable (Stalmans et al. 2019). Sable have not benefitted from translocations into Gorongosa, as buffalo have in limited numbers (approximately 200) (Stalmans et al. 2019). Sable population recovery may have benefitted from slightly shorter gestation periods (8 months) and calving intervals (9 months) than both zebra (gestation = 12 months; calving interval = 13 months) and buffalo (gestation = 11 months; calving interval = 20–26 months), with sable and buffalo births more seasonally constrained than those of zebra (Carmichael et al. 1977; Sekulic 1978; Grobler 1981; Grange et al. 2004; Ryan et al. 2007; Ogutu et al. 2014). The non-logistic growth of all three species, however, suggests that external factors have influenced expansion of post-war range size by tempering population growth rates (Stalmans et al. 2019).

The sparse coverage of aerial surveys immediately post-war may potentially have underestimated the residual population of sable, and thus range size, after the conflict. The aerial surveys of Gorongosa have been concentrated on the rift valley floor where most of the animal biomass in the park is concentrated, with largely open habitat making for easier detection of grazer species. However, sable often occupy savanna woodland in other parts of southern Africa, which may make them less easily detectable from aerial surveys, exacerbating the inaccuracy in estimates from the surveys immediately post-war (Chirima et al. 2013; Owen-Smith et al. 2013).

We were unable to use detection rates to improve model accuracy, as spatial covariates (specifically time or distance) were not recorded for the game counts. The data from the aerial surveys, despite the discrepancies between sampling regimes, represent an improvement in the widely practiced use of museum data to build models of ecological niches (see for example Elith et al. 2006; Schoeman et al. 2013; Bloom et al. 2018; Kessler et al. 2022). The data used in our study arise from systematic surveys over Gorongosa and are less prone to gaps in data that arise from the haphazard and opportunistic sampling that characterize museum collections. While we acknowledge potential gaps in the data associated with detectability and variable coverage between years, the sheer magnitude of change in range size between the pre- and post-war period are unlikely to be due solely to sampling discrepancies. Data were pooled into one of two periods, standardizing the area covered as surveys from 2014 onward were matched to survey routes pre-war. Moreover, through spatial filtering we standardized the minimum distance separating GPS localities, removing clustering bias associated with differential sampling regimes. Observations of shifts in ecological niche characteristics and changes in range size are thus reflective of patterns across the whole duration of each period, and not only a handful of aerial surveys in shorter-time periods that may be more sensitive to differences in sampling coverage.

### Interspecific niche dynamics

The war-induced species-specific changes in ecological niche characteristics and range size have substantially altered interspecific niche overlap patterns. The civil war, through altering relative abundances and subsequently range size and distribution patterns, has resulted in greater spatial niche partitioning between these species. Under the pre-war scenario, reciprocal pairwise ecological similarity was observed for all species pairs, whereas this only remained the same for the sable-waterbuck pairing in the post-war period. Our findings support our second hypothesis regarding interspecific overlap patterns, predicting that the decreases in range size for buffalo and zebra would lead to decreased spatial niche overlap in the post-war period. The decreases in spatial niche overlap were significant in the post-war period, with non-reciprocal interspecific ecological dissimilarity observed between several species pairs. These results suggest that despite the recovery in mammalian biomass to almost pre-war levels, ecological complexities between species have been altered.

The results of the niche similarity tests indicated that both buffalo and zebra niches were nested within both the sable and waterbuck niches. Ecological dissimilarity has likely resulted from the contrasting fortunes of species-specific changes in range size, given the high niche similarity displayed by both buffalo and zebra towards both waterbuck and sable, contrasting with the ecological dissimilarity displayed in the other direction of these relationships. The spatial niche partitioning observed as a result of severe anthropogenic disturbance in our study contrasts with the findings of several recent studies, where human-driven niche compression of coexisting species was observed, resulting in higher than expected niche overlap across taxa including birds, fish, and mammalian carnivores (Lara et al. 2015; Smith et al. 2018; Burdon et al. 2020; Manlick and Pauli 2020). Anthropogenic homogenization of available resources has been a key driver of decreased trophic niche partitioning in carnivore communities (Smith et al. 2018; Manlick and Pauli 2020; Sévêque et al. 2020). However, anthropogenic alterations to the landscape leading to increased niche partitioning has been observed in previous studies, but is considerably less frequent (Pereira et al. 2012; Cruz et al. 2015). The context of our results represents a more unique scenario, where anthropogenic disturbance constituted a severe but singular event, rather than the prolonged and ever-present disturbances focused on in much of the literature. Moreover, the armed conflict in Mozambique reduced mammal populations within Gorongosa through top-down hunting for bushmeat, resulting in a mass mortality event, and not through bottom-up alterations of the landscape with subsequent influences on resource availability.

The relationship between dietary niche partitioning and spatial niche partitioning among herbivores in Gorongosa has not been fully and coherently assessed. Similarly to our results, tests of dietary similarity in Gorongosa revealed all grazer species analysed in this study overlapped significantly with waterbuck in terms of dietary composition (Pansu et al. 2019). In the Kruger National Park (KNP), wildebeest (*Connochaetes taurinus*) and zebra showed high spatial overlap of home ranges, however they showed greater distinction in diet (Owen-Smith et al. 2015). High dietary overlap between zebra and buffalo in KNP was mediated by spatial separation, whereas high spatial overlap between zebra and sable in the same system was mediated by a greater degree of dietary separation (Macandza et al. 2012).

Pansu et al. (2019) found high dietary overlap between grazers in landscapes with homogeneous resources, whereas interspecific dietary partitioning between browsers was more pronounced in areas with greater resource heterogeneity, highlighting the influence of habitat complexity in niche overlap. While it is possible that the high dietary overlap in Gorongosa may be facilitated by the emergence of greater spatial niche partitioning between species in the post-war period, the results of our study, given the mismatch in scale and resolution, cannot be used in conjunction with the relevant dietary studies to generate a satisfactory picture of the diet-space interplay of niche partitioning. This is exacerbated by the inaccessibility by road to over 60% of Gorongosa, preventing fine-scale characterization of habitat-specific resource availability over much of the area surveyed in the game counts.

Predation pressure may be more important than resource availability in determining spatial niche partitioning between large African grazer species (Owen-Smith 2019b). Lions in particular may choose areas with preferred prey over areas with greater prey biomass (Hayward and Slotow 2009) however prey preferences may also change according to availability (Valeix et al. 2012). At present, lions in Gorongosa prefer to feed on highly abundant smaller-bodied herbivores such as reedbuck and warthog (Bouley et al. 2018) though historical records suggest that lions used to prey on larger ungulates such as wildebeest and buffalo (Tinley 1977). The stability in patterns of niche overlap observed in our findings is thus contingent not only on population recoveries of the focal grazer species, but also on the top-down pressure exerted by growing populations of predators in Gorongosa strengthened by reintroduction efforts.

High spatial overlap between prey species may dilute the effect of predation on the population of a single species (Hamilton 1971; Van Valen 1974; Schmitt et al. 2014). However, species such as sable may reduce predation risk through spatial avoidance of areas with high densities of important prey species (Chirima et al. 2013). How behavioural shifts, if observed, to growing predation pressure may influence interspecific spatial niche overlap is an interesting scientific prospect and a serious consideration for effective management. Experimental manipulation of vocal and olfactory predator signals in Gorongosa led to greater occupancy by bushbuck of habitats with high woody cover, more characteristic of the species’ generally observed habitat preferences, reversing the locally observed prevalence in open floodplains (Atkins et al. 2019). We were unable to determine the effects of decreased predation pressure on distribution patterns of the four focal grazer species, due to a dearth of historical georeferenced localities for predator species, as well as the unsuitability of a correlative ENM approach for such a problem.

The diffuse predation pressure was a critical aspect in our decision to base our predictions on a variation of the abundance-occupancy hypothesis, which proposes that species which are more abundant tend to occupy more sites (Gaston et al. 2000). Our slight variation on this hypothesis related to changes in range size within species that have undergone substantial changes in abundance because of the armed conflict in central Mozambique, and how this would influence interspecific spatial patterns. The results of our study thus characterize changes to patterns of spatial niche overlap that emerge over a recovery period that is now twice the duration of the conflict that so severely reduced mammalian populations in Gorongosa.

Our findings present several interesting implications for further assessment, as well as several caveats to consider when interpreting our results. The degree to which our results represent a fundamental alteration to niche partitioning patterns or an intermediary step in the recovery of pre-war spatial dynamics cannot be understood fully at present. By pooling our data into periods, we have sacrificed the resolution in understanding changes to patterns over shorter timeframes in order to assess how these patterns have altered given the subsequent recovery period. The period of time elapsed may still be insufficient for species abundances to sufficiently recover and repopulate historical niche space. Additionally, it is beyond the scope of our study to test changes in behavioural mechanisms that may influence spatial niche partitioning between these grazer species. These questions, while intriguing, are unsuitable for a correlative ENM procedure, and the lack of time-specific field observations for these species over the study period are a critical limitation in this aspect.

Our findings also present interesting implications for understanding the interplay between neutral and deterministic processes shaping community assembly. The influence of stochastic processes such as extinction and dispersal on community assembly have long been proposed in ecology (Hubbell 2001; Rosindell et al. 2012), with case studies on bacteria and grasshopper communities highlighting the potential utility of these frameworks (Rominger et al. 2009; Caruso et al. 2011; Langenheder and Székely 2011). A niche-based hypothesis may posit that buffalo, as large bodied bulk-feeders, and zebra, as hindgut fermenters that can digest more fibrous plant parts, may over time competitively displace waterbuck, which have shown higher ectoparasite load and reduced digestive efficiency in the savannah environments of Gorongosa (Becker et al. 2021). Conversely, if stochastic processes are more important, the favoured population growth of waterbuck, and to a lesser degree sable, may maintain a community where both species are more numerous than the competitively superior zebra and buffalo. Quantifying whether future patterns of spatial niche overlap converge towards the historically observed patterns or diverge in a direction associated with greater niche expansion from species which have grown in abundance post-war, may provide an interesting insight into the interplay between neutral and deterministic processes in community assembly.

## Conclusions

Establishing how species distributions have been altered as a result of anthropogenic change provides valuable information on how specific ecological and evolutionary processes are altered by disturbance events. Our findings suggest that despite biomass recovery over a period that is now twice the duration of the armed conflict that resulted in drastic population declines, species-specific changes in distribution associated with differential fortunes in population growth have significantly altered patterns of interspecific spatial niche overlap among four grazer species. The alteration of spatial overlap patterns of sympatric species between pre- and post-war scenarios, highlights how human interference can erode the pressures of selection that account for niche partitioning. Our findings present several implications for future studies on the niche dynamics of grazers in the Gorongosa system, and whether the patterns we observed represent fundamental alterations to patterns of niche similarity, or simply an intermediary step towards the re-establishment of historical precedents.

## Supplementary Information

Below is the link to the electronic supplementary material.

Supplementary file1 (DOCX 26 KB)

## Data Availability

Raw data already available online from cited dataset, however, cleaned datasets can be provided for the journal upon request.

## Code availability

Non-Applicable.
