# Supplementary material for: Anthropogenically driven spatial niche partitioning in a large herbivore assemblage
Source: Oecologia. 2023 Mar 1;201(3):797–812. doi: 10.1007/s00442-023-05342-9 (PMC10038942; doi:10.1007/s00442-023-05342-9)
Supplement: Supplementary file 1 — Supplementary file1 (DOCX 26 KB) [file 442_2023_5342_MOESM1_ESM.docx]

Table S1. Aerial survey methodology for the years where georeferenced localities for animals were taken.

| Year of count | Season | Month | Type of count | Platform | Strip width (m) | Percentage of Gorongosa covered |
| --- | --- | --- | --- | --- | --- | --- |
| 1969 | Dry | November | Full count | Fixed-wing | 1 000 | 64.7 |
| 1970 | Wet | January | Full count | Fixed-wing | 1 000 | 64.7 |
| 1972 | Dry | October | Full count | Fixed-wing | 1 000 | 64.7 |
| 1994 | Dry | June | Sample count | Fixed-wing | 255 | 2.9 |
| 1997 | Dry | October | Sample count | Fixed-wing | 255 | 5.2 |
| 2000 | Dry | October | Sample count | Helicopter | 500 | 11.6 |
| 2001 | Dry | November | Sample count | Helicopter | 500 | 24.4 |
| 2002 | Dry | November | Sample count | Helicopter | 500 | 14.6 |
| 2004 | Dry | October | Sample count | Fixed-wing | 303 | 9.6 |
| 2007 | Dry | November | Sample count | Helicopter | 500 | 21.9 |
| 2010 | Dry | November | Sample count | Helicopter | 500 | 22.5 |
| 2012 | Dry | May | Sample count | Helicopter | 500 | 23.8 |
| 2014 | Dry | October | Full count | Helicopter | 500 | 49.9 |
| 2016 | Dry | October | Full count | Helicopter | 500 | 53.9 |
| 2018 | Dry | October | Full count | Helicopter | 500 | 58.9 |

Table S2. Worldclim variables used in the PCA for building ENMs.

| **Variable code** | **Meaning** |
| --- | --- |
| BIO 1 | Annual mean temperature |
| BIO 2 | Mean diurnal range |
| BIO 3 | Isothermality |
| BIO 4 | Temperature seasonality |
| BIO 5 | Maximum temperature of warmest month |
| BIO 6 | Minimum temperature of coldest month |
| BIO 7 | Annual temperature range |
| BIO 10 | Mean temperature of warmest quarter |
| BIO 11 | Mean temperature of coldest quarter |
| BIO 12 | Mean annual precipitation |
| BIO 13 | Precipitation of wettest month |
| BIO 14 | Precipitation of driest month |
| BIO 15 | Precipitation seasonality |
| BIO 16 | Precipitation of wettest quarter |
| BIO 17 | Precipitation of driest quarter |

Table S3. Loadings for each bioclimatic variable into each component generated by the PCA. Variables that contribute highly to a component are highlighted in bold. Cumulative variance of the original Worldclim data explained by the addition of each component is included at the bottom of the table.

| **Variable** | **PC 1** | **PC 2** | **PC 3** | **PC 4** |
| --- | --- | --- | --- | --- |
| BIO 1 | 0.234 | -0.349 | -0.220 | 0.089 |
| BIO 2 | **0.294** | 0.084 | -0.200 | 0.197 |
| BIO 3 | -0.101 | **-0.467** | 0.286 | **0.526** |
| BIO 4 | **0.273** | 0.219 | -0.177 | 0.179 |
| BIO 5 | **0.295** | 0.027 | -0.323 | -0.081 |
| BIO 6 | -0.205 | **-0.403** | 0.061 | -0.282 |
| BIO 7 | **0.284** | 0.181 | -0.244 | 0.059 |
| BIO 10 | 0.245 | -0.307 | -0.239 | 0.197 |
| BIO 11 | 0.148 | **-0.484** | -0.184 | 0.034 |
| BIO 12 | **-0.299** | 0.040 | -0.241 | 0.234 |
| BIO 13 | **-0.278** | 0.126 | -0.027 | **0.501** |
| BIO 14 | **-0.265** | -0.168 | -0.334 | -0.412 |
| BIO 15 | **0.279** | 0.071 | **0.484** | -0.010 |
| BIO 16 | **-0.283** | 0.164 | -0.204 | 0.177 |
| BIO 17 | **-0.295** | 0.053 | -0.305 | 0.090 |
| Cumulative variance (%) | **70.258** | **91.260** | **95.059** | **97.316** |

Table S4. Training AUC scores for pre- and post-war ENMs constructed for buffalo. Highest AUC values are highlighted in bold. Models were constructed using unique combinations of three variations of regularization multiplier (RM = 1; 2; or 4) and three different feature class combinations.

| Model | Pre-War AUC | Post-War AUC |
| --- | --- | --- |
| RM = 1; FC = lq | 0.694 | 0.832 |
| RM = 1; FC = lqp | **0.710** | **0.835** |
| RM = 1; FC = q | 0.672 | 0.806 |
|  |  |  |
| RM = 2; FC = lq | 0.693 | 0.829 |
| RM = 2; FC = lqp | 0.710 | 0.832 |
| RM = 2; FC = q | 0.670 | 0.807 |
|  |  |  |
| RM = 4; FC = lq | 0.690 | 0.821 |
| RM = 4; FC = lqp | 0.706 | 0.827 |
| RM = 4; FC = q | 0.665 | 0.807 |

Abbreviations for feature class labels: lq = linear and quadratic; lqp = linear, quadratic and product; q = quadratic.

Table S5. Training AUC scores for pre- and post-war ENMs constructed for sable. Highest AUC values are highlighted in bold. Models were constructed using unique combinations of three variations of regularization multiplier (RM = 1; 2; or 4) and three different feature class combinations.

| Model | Pre-War AUC | Post-War AUC |
| --- | --- | --- |
| RM = 1; FC = lq | 0.724 | 0.657 |
| RM = 1; FC = lqp | **0.726** | **0.662** |
| RM = 1; FC = q | 0.573 | 0.597 |
|  |  |  |
| RM = 2; FC = lq | 0.723 | 0.654 |
| RM = 2; FC = lqp | 0.721 | 0.659 |
| RM = 2; FC = q | 0.568 | 0.599 |
|  |  |  |
| RM = 4; FC = lq | 0.717 | 0.646 |
| RM = 4; FC = lqp | 0.711 | 0.654 |
| RM = 4; FC = q | 0.558 | 0.595 |

Abbreviations for feature class labels: lq = linear and quadratic; lqp = linear, quadratic and product; q = quadratic.

Table S6. Training AUC scores for pre- and post-war ENMs constructed for waterbuck. Highest AUC values are highlighted in bold. Models were constructed using unique combinations of three variations of regularization multiplier (RM = 1; 2; or 4) and three different feature class combinations.

| Model | Pre-War AUC | Post-War AUC |
| --- | --- | --- |
| RM = 1; FC = lq | 0.790 | 0.616 |
| RM = 1; FC = lqp | **0.794** | **0.620** |
| RM = 1; FC = q | 0.600 | 0.571 |
|  |  |  |
| RM = 2; FC = lq | 0.790 | 0.616 |
| RM = 2; FC = lqp | 0.793 | 0.619 |
| RM = 2; FC = q | 0.603 | 0.569 |
|  |  |  |
| RM = 4; FC = lq | 0.788 | 0.615 |
| RM = 4; FC = lqp | 0.792 | 0.617 |
| RM = 4; FC = q | 0.606 | 0.564 |

Abbreviations for feature class labels: lq = linear and quadratic; lqp = linear, quadratic and product; q = quadratic.

Table S7. Training AUC scores for pre- and post-war ENMs constructed for zebra. Highest AUC values are highlighted in bold. Models were constructed using unique combinations of three variations of regularization multiplier (RM = 1; 2; or 4) and three different feature class combinations.

| Model | Pre-War AUC | Post-War AUC |
| --- | --- | --- |
| RM = 1; FC = lq | 0.679 | **0.827** |
| RM = 1; FC = lqp | 0.684 | 0.820 |
| RM = 1; FC = q | 0.651 | 0.723 |
|  |  |  |
| RM = 2; FC = lq | 0.681 | 0.817 |
| RM = 2; FC = lqp | **0.685** | 0.810 |
| RM = 2; FC = q | 0.650 | 0.723 |
|  |  |  |
| RM = 4; FC = lq | 0.680 | 0.811 |
| RM = 4; FC = lqp | 0.683 | 0.787 |
| RM = 4; FC = q | 0.645 | 0.720 |

Abbreviations for feature class labels: lq = linear and quadratic; lqp = linear, quadratic and product; q = quadratic.
